# Supplementary material for: Differentiation of IL-26+ TH17 intermediates into IL-17A producers via epithelial crosstalk in psoriasis
Source: Nat Commun. 2023 Jun 30;14:3878. doi: 10.1038/s41467-023-39484-4 (PMC10313793; doi:10.1038/s41467-023-39484-4)
Supplement: Supplementary file 1 — Supplementary Information [file 41467_2023_39484_MOESM1_ESM.pdf]

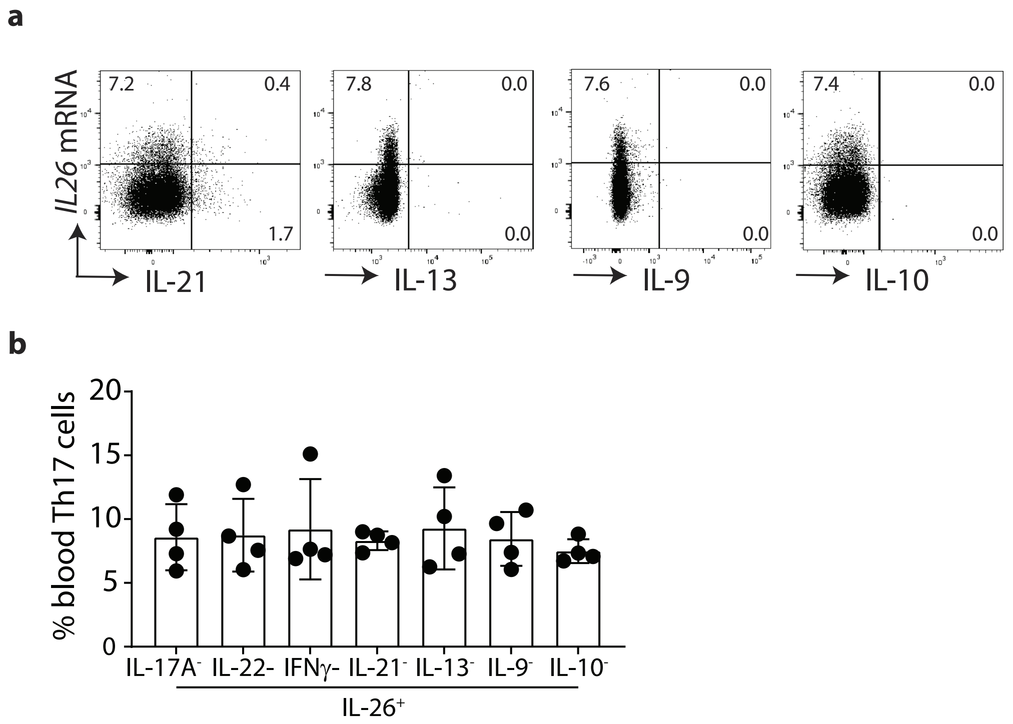

Supplementary Figure 1. IL-26⁺ IL17A⁻ Th17 cells do not produce other Th17-derived cytokines

a, Flow-FISH analysis of the expression of IL-26 mRNA, and IL-21, IL-13, IL-9, and IL-10 protein in blood-isolated memory TH17 cells. Data from one representative donor is shown.
b, Frequencies of IL-26 positive cells and negative for other cytokines among blood memory TH17 cells. Data are mean±SD, (n=4 donors).
Source data are provided as a Source Data file.

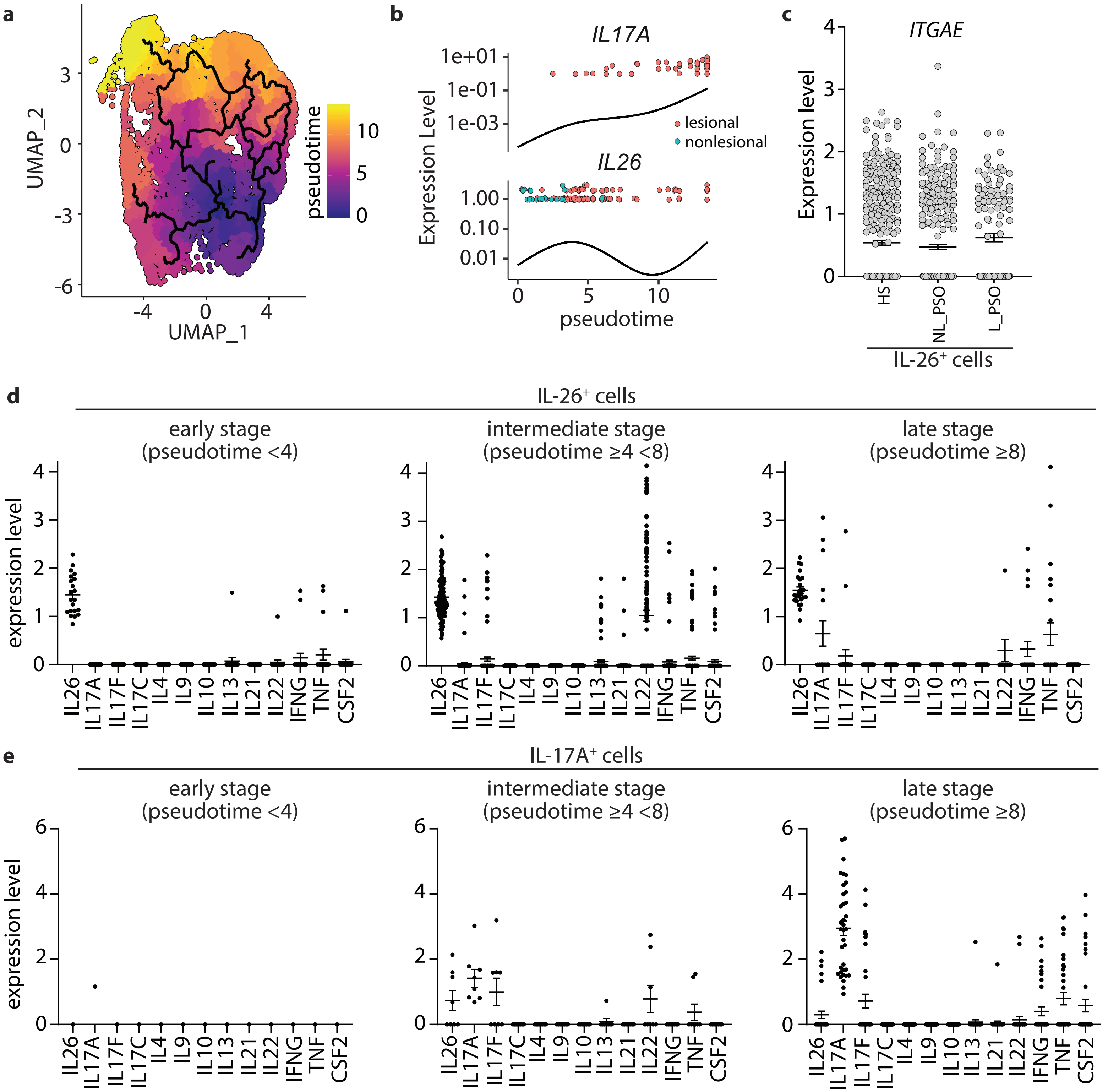

Suppl Figure 2. IL-26 production in psoriatic skin occurs early, whereas other Th17-derived cytokines such as IL-17A, IL-22, IL-21 and GM-CSF are induced at later timepoints.

a, UMAP projection of the single cell transcriptomes of dermal CD4 T cells from nonlesional (left) and lesional (right) skin of 3 psoriasis patients colored according to the inferred pseudotime.
Solid lines are trajectories of gene expression changes learned by Monocle3.
b, Dynamics of IL-17A and IL-26 gene expression in nonlesional and lesional dermal CD4 T cells over pseudotime.
Solid lines show the expression average.
c, Expression of ITGAE (CD103) by IL26+ T cells from healthy (n=379), non lesional (n=304 cells), and lesional (n=116 cells) psoriatic skin. Data represent mean±SEM.
d-e, Expression level of different cytokines by IL-26 positive (d) and IL-17A positive (e) dermal CD4 T cells from the skin of 3 psoriasis patients at early (left, n=21 IL26+ cells and 1 IL17A+ cells), intermediate (middle, n=121 IL26+ cells and 8 IL17A+ cells), and late (right, n=24 IL26+ cells and 36 IL17A+ cells) stage of differentiation according to the inferred pseudotime. Data represent mean±SEM.
Source data are provided as a Source Data file.

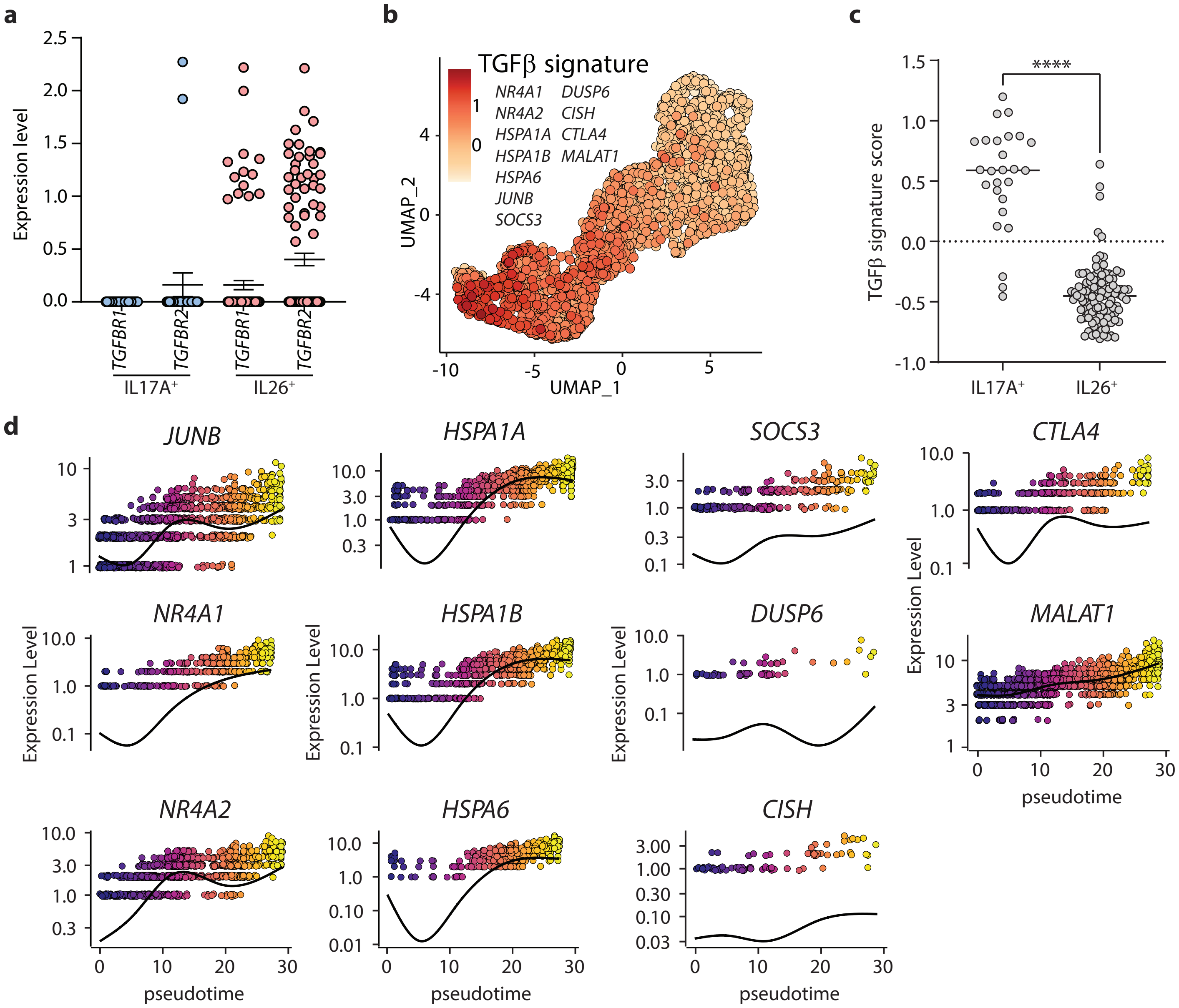

Supplementary Figure 3. Transition from IL-26 to IL-17A producing T cells in psoriatic skin lesions display TGF-β imprinting

a, Expression of TGFBR1 and TGFBR2 by IL17A+ (n=26 cells) and IL26+ T cells (n=107) from lesional psoriatic skin. Data represent mean±SEM.
b, UMAP projection of the single cell transcriptomes of dermal CD4 T cells as in Figure 4 colored according to the expression level of the TGF-β signature genes.
c, Quantification of TGF-β signature score in IL-17A- (n=26 cells) and IL-26-single positive cells (n=107). Bars represent mean values. Data were statistically analyzed using two-tailed unpaired Student's t-test. ****p<0.0001.
d, Dynamics of the TGF-β signature genes expression in dermal CD4 T cells over pseudotime. Solid lines show the expression average.
Source data are provided as a Source Data file.

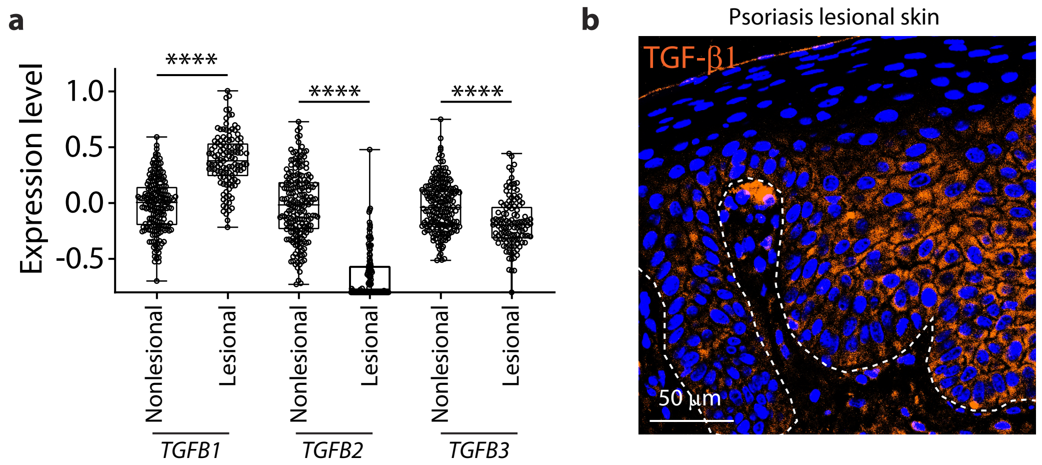

Supplementary Figure 4. Increased expression of TGFB1, but not TGFB2 nor TGFB3 in keratinocytes of the basal layers in psoriatic skin

a, Expression level of TGFB1, TGFB2, and TGFB3 in nonlesional (n=199) and lesional (n=120) skin from psoriasis patients using the SSF Bioinformatics hub.
Data are represented as a box plot which bounds extends from the 25th to 75th percentiles, a middle line is plotted at the median, and whiskers go from the minimal to maximal value.
Data were statistically analyzed using one-way ANOVA followed by Tukey's multiple comparisons test. ****p<0.0001.
b, Confocal microscopy images of lesional psoriatic skin representative of 3 patients stained for TGF-β1 (orange).
Dashed line delineates the dermo-epidermal junction.
Source data are provided as a Source Data file.

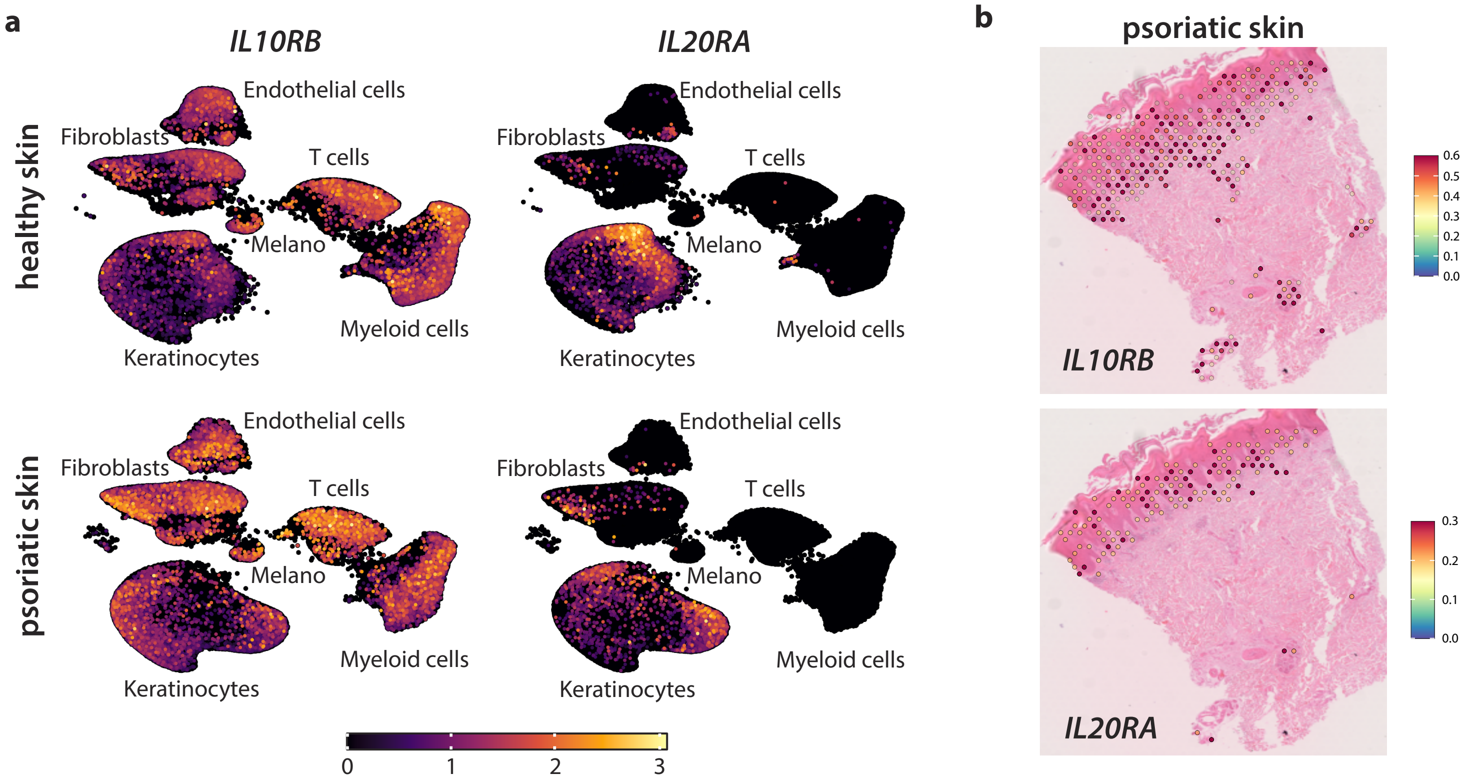

Supplementary Figure 5. Healthy and psoriatic keratinocytes express the IL-26 receptor

a, UMAP projection of the single cell transctiptomes of cells from the skin of 5 healthy donors (top) and 3 psoriasis patients (bottom) colored according to the expression level of IL10RB (left) and IL20RA (right). **b**, Spatial mapping of IL10RB (left) and IL20RA (right) expression in the lesional skin of a psoriasis patient.

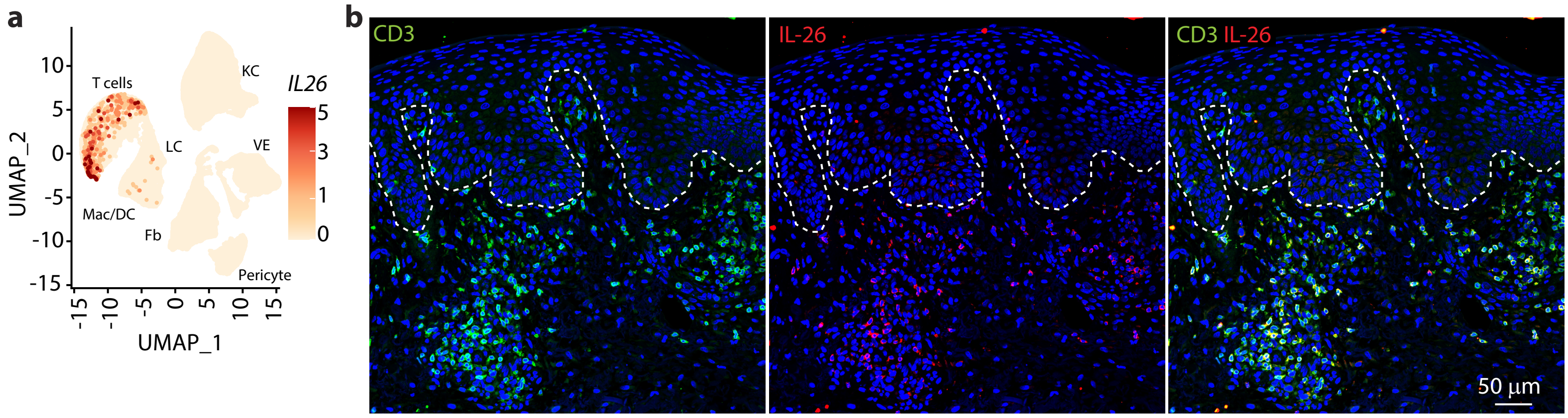

Supplementary Figure 6. IL-26 is predominantly expressed by T cells in psoriatic plaques

a, UMAP projection of the single cell transcriptomes of total cells from lesional skin of 3 psoriasis patients colored according to the expression level of IL26.
b, Confocal microscopy images of lesional psoriatic skin representative of 5 patients stained for CD3 (green) and IL-26 (red). Dashed line delineates the dermo-epidermal junction.

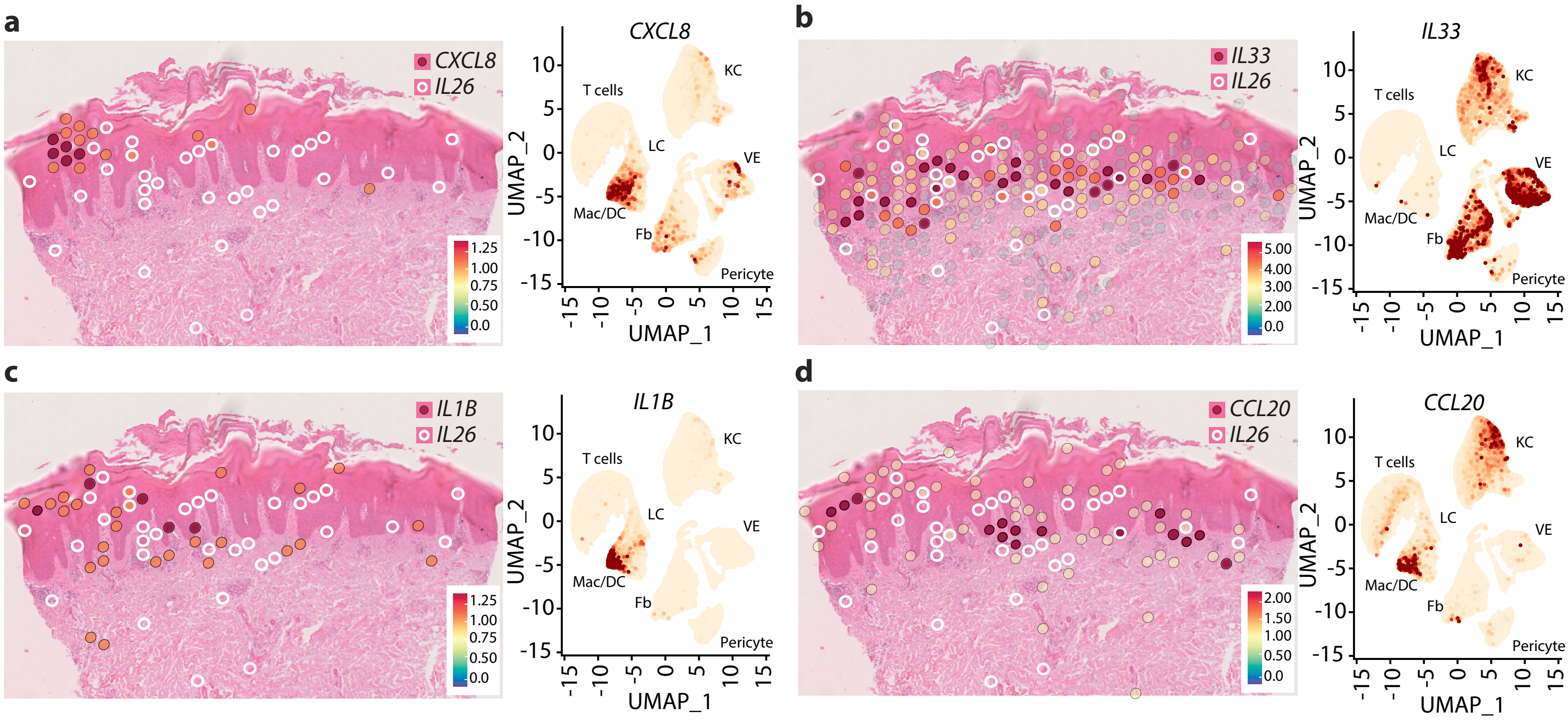

Supplementary Figure 7. IL-1β, IL-33, and CCL20 are expressed by dermal monocytes/macrophages and fibroblasts, and do not co-localize with IL-26-expressing T cells

a-d, Spatial mapping of CXCL8 (a), IL33 (b), IL1B (c), and CCL20 (d) expression in the lesional skin of a psoriasis patient (left) and UMAP projection of the single cell transcriptomes of total cells from lesional skin of 3 psoriasis patients colored according to the expression level of each gene (right). White circles in the spatial image show IL26+ spots.

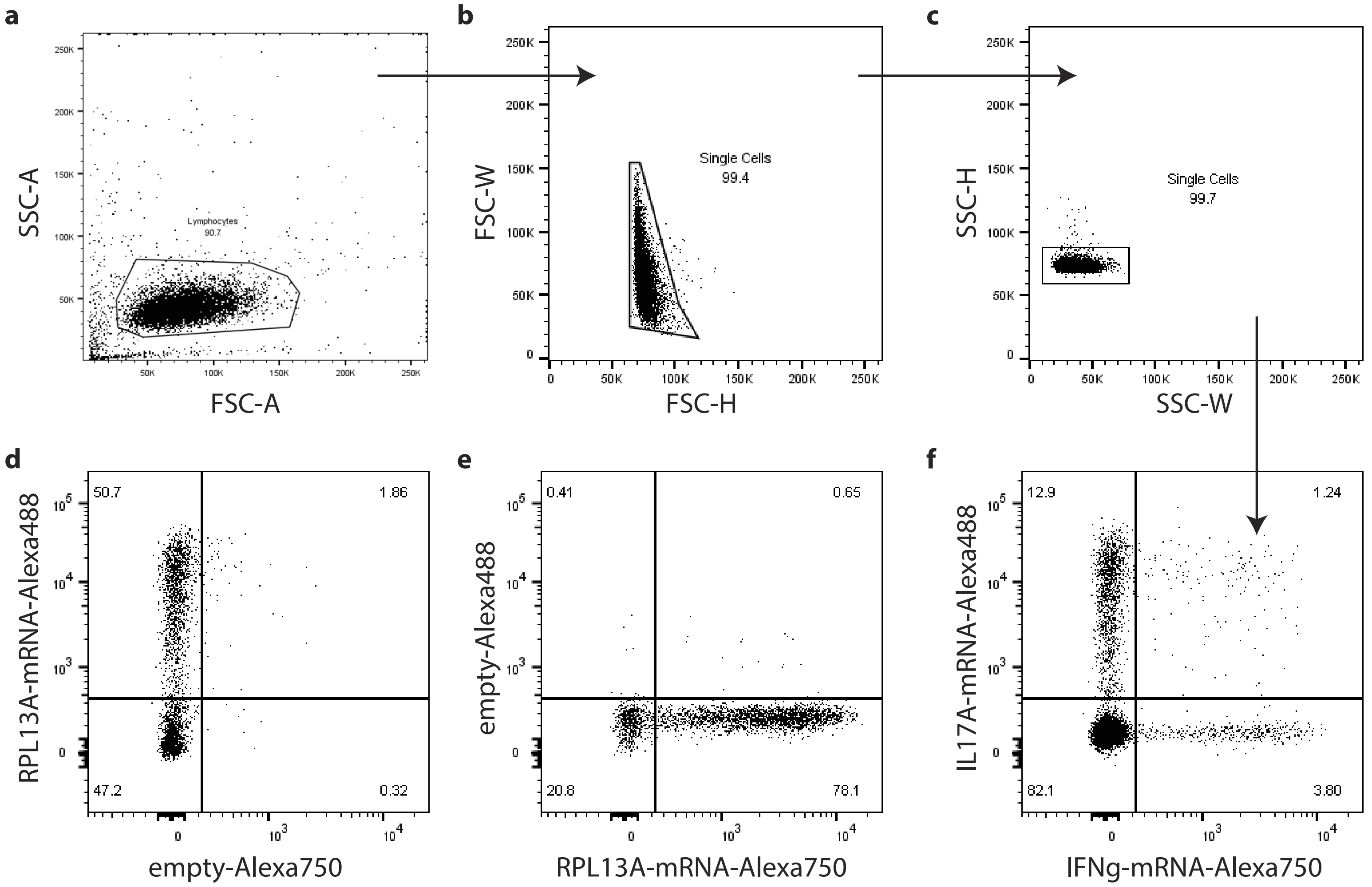

Supplementary Figure 8. Gating strategy for Flow-FISH analysis

a, Live blood-isolated Th17 cells (or in vitro differentiated Th17 cells) were gated based on SSC and FSC area parameters as an homogenous population.
b-c, doublet cells were then excluded using the height and width of FSC and SSC respectively.
d-e, The Flow-FISH procedure was verified by assessing the housekeeping RPL13A mRNA expression using positive control probes labeled with Alexa488 or Alexa750.
f, The mRNA expression of cytokines of interest were measured (e.g. IL17A, INFG).

Supplementary Table 1. Human samples and datasets used in this study

Number of patients	Age in years Median (Range)	Material	Ethics/Consent	Figures	Reference
5 psoriasis skin samples	36 (32-55)	Biobanked FFPE material	CER-VD 2020-02204	Fig.6c; Fig.7; SuppFig.4b; SuppFig.5b, SuppFig.6b	NA
3 healthy skin samples	43 (30-52)	Fresh residual material from excisional surgery	CER-VD 2020-02204	Fig.6f	NA
15 healthy blood samples	unknown	Buffy coats	Interregional Blood Transfusion Center, project 85	Fig.1 to 3; SuppFig.1, SuppFig.8	NA
134 psoriasis skin samples	See publication	Re-analysis of published microarray datasets	See publication	Fig.6a; SuppFig.4a	(36)
3 psoriasis skin samples	See publication	Re-analysis of published single-cell RNAseq datasets	See publication	Fig.4; Fig.6b; SuppFig.2, SuppFig.3, SuppFig.5a, SuppFig.6a, SuppFig.7	(24)
10 psoriasis	See publication	Re-analysis of published single-cell RNAseq and TCRseq datasets	See publication	Fig.5	(25)
