# Supplementary material for: Differentiation of IL-26+ TH17 intermediates into IL-17A producers via epithelial crosstalk in psoriasis
Source: Nat Commun. 2023 Jun 30;14:3878. doi: 10.1038/s41467-023-39484-4 (PMC10313793; doi:10.1038/s41467-023-39484-4)
Supplement: Supplementary file 3 — Reporting Summary [file 41467_2023_39484_MOESM3_ESM.pdf]

Corresponding author(s): Jeremy Di Domizio and Michel Gilliet

Last updated by author(s): Jun 2, 2023

Reporting Summary

Nature Portfolio wishes to improve the reproducibility of the work that we publish. This form provides structure for consistency and transparency in reporting. For further information on Nature Portfolio policies, see our Editorial Policies and the Editorial Policy Checklist.

Statistics

For all statistical analyses, confirm that the following items are present in the figure legend, table legend, main text, or Methods section.

n/a	Confirmed	
☐	☒	The exact sample size (*n*) for each experimental group/condition, given as a discrete number and unit of measurement
☐	☒	A statement on whether measurements were taken from distinct samples or whether the same sample was measured repeatedly
☐	☒	The statistical test(s) used AND whether they are one- or two-sided<br>*Only common tests should be described solely by name; describe more complex techniques in the Methods section.*
☒	☐	A description of all covariates tested
☒	☐	A description of any assumptions or corrections, such as tests of normality and adjustment for multiple comparisons
☐	☒	A full description of the statistical parameters including central tendency (e.g. means) or other basic estimates (e.g. regression coefficient) AND variation (e.g. standard deviation) or associated estimates of uncertainty (e.g. confidence intervals)
☐	☒	For null hypothesis testing, the test statistic (e.g. *F*, *t*, *r*) with confidence intervals, effect sizes, degrees of freedom and *P* value noted<br>*Give P values as exact values whenever suitable.*
☒	☐	For Bayesian analysis, information on the choice of priors and Markov chain Monte Carlo settings
☒	☐	For hierarchical and complex designs, identification of the appropriate level for tests and full reporting of outcomes
☐	☒	Estimates of effect sizes (e.g. Cohen's *d*, Pearson's *r*), indicating how they were calculated

Our web collection on statistics for biologists contains articles on many of the points above.

Software and code

Policy information about availability of computer code

Data collection	Data were collected in part with CaseCenter™ slide management system, ZEN 3.2 Imaging Software, SkanIt™ Software, QuantStudio (5, 6 or 7), BD FACSDiva™ Software v. 6.1.3
Data analysis	Data were anlyzed using QuantCenter™ image analysis, ZEN 3.2 Imaging Software, FlowJo_v10.7.1 ,GraphPad PRISM 9, Microsoft Excel v2208, R Studio 2022.07.2, R version 4.2.2., R package Seurat v4.0, R package Monocle 3, R package SpatialExperiment v1.4

For manuscripts utilizing custom algorithms or software that are central to the research but not yet described in published literature, software must be made available to editors and reviewers. We strongly encourage code deposition in a community repository (e.g. GitHub). See the Nature Portfolio guidelines for submitting code & software for further information.

Data

Policy information about availability of data

All manuscripts must include a data availability statement. This statement should provide the following information, where applicable:

- Accession codes, unique identifiers, or web links for publicly available datasets
- A description of any restrictions on data availability
- For clinical datasets or third party data, please ensure that the statement adheres to our policy

Microarray transcriptional profiles generated by Fyhrquist N., et al. (39) are available via the Skin Science Foundation's bioinformatics hub [https://biohub.skinsciencefoundation.org]. Previously published single-cell RNA and TCR sequencing datasets by Reynolds G., et al. (24) and Zhang B., et al. (25), are deposited at ArrayExpress [www.ebi.ac.uk/arrayexpress/experiments/E-MTAB-8142] and at the European Genome-phenome Archive (EGA) [https://ega-

archive.org/datasets/EGAD00001010106], respectively. Spatial transcriptomic datasets generated by Schabitz A, et al., (36) (GSE206391 – [https://www.ncbi.nlm.nih.gov/geo/query/acc.cgi?acc=GSE206391]) and by us (GSE173706 – [https://www.ncbi.nlm.nih.gov/geo/query/acc.cgi?acc=GSE173706]) are deposited at GEO data repository. Source data are provided with this paper.

Human research participants

Policy information about studies involving human research participants and Sex and Gender in Research.

Reporting on sex and gender	The information about sex and gender has not been collected in this study.
Population characteristics	Defined in Supplementary Table 1
Recruitment	Fresh leftover surgical tissue from healthy skin and biobanked FFPE psoriatic skin tissue stored in the Swiss Biobanking Platform (SBP)-accredited Dermatology biobank were obtained from consented patients
Ethics oversight	Studies were approved by the institutional review boards and the local ethics committee of the Lausanne University Hospital CHUV, Switzerland, in accordance with the Helsinki Declaration and were reviewed by the ethical committee board of the canton of Vaud, Switzerland (CER-VD 2020-02204).

Note that full information on the approval of the study protocol must also be provided in the manuscript.

Field-specific reporting

Please select the one below that is the best fit for your research. If you are not sure, read the appropriate sections before making your selection.

[x] Life sciences [ ] Behavioural & social sciences [ ] Ecological, evolutionary & environmental sciences

For a reference copy of the document with all sections, see nature.com/documents/nr-reporting-summary-flat.pdf

Life sciences study design

All studies must disclose on these points even when the disclosure is negative.

Sample size	We have used at least three biological replicates for each experiment - unless stated otherwise. This is consistent with previous studies and accounts for biological variability observed in similar experiments as demonstarted by David BH, Cytometry B Clin Cytom . 2013 Sep-Oct;84(5):329-37. doi: 10.1002/cyto.b.21116. where they conclude that three to four replicates are sufficient for most flow cytometric assays and instrument combinations.
Data exclusions	No data was excluded.
Replication	Experimental findings were reliably reproduced. The number (n) of biological replicates is indicated as an exact number in the figure legends.
Randomization	The experiments were randomized by splitting blood or keratinocyte donor cells into random wells before adding stimuli/treatments.
Blinding	The investigators in charge of the readouts assessment were blinded as experimental samples (antibody-stained cells, cell supernatants, tissue sections) were prepared and coded by other investigators.

Reporting for specific materials, systems and methods

We require information from authors about some types of materials, experimental systems and methods used in many studies. Here, indicate whether each material, system or method listed is relevant to your study. If you are not sure if a list item applies to your research, read the appropriate section before selecting a response.

Materials & experimental systems

n/a	Involved in the study
[ ]	[x] Antibodies
[ ]	[x] Eukaryotic cell lines
[x]	[ ] Palaeontology and archaeology
[x]	[ ] Animals and other organisms
[x]	[ ] Clinical data
[x]	[ ] Dual use research of concern

Methods

n/a	Involved in the study
[x]	[ ] ChIP-seq
[ ]	[x] Flow cytometry
[x]	[ ] MRI-based neuroimaging

Antibodies

Antibodies used

anti-human IL-9 PeCy7 (BioLegend, #507611)
anti-human IL-10 PE (BD Biosciences, #559330)
anti-human IL-13 BV421 (BioLegend, #501915)
anti-human IL-21 PE (BioLegend, #513004)
goat anti-human IL-26/AK155 (R&D systems, #AF1375)
blocking anti-IL-26 (Patented Clone 84)
anti-IL-10R2 (R&D Systems Clone # 90220)
anti-IL-20R1 (R&D Systems Clone # 173707)
mouse anti-human TGF beta-1 (Thermo Fisher Scientific, # MA5-16949)
anti-human IL-4 (eBioscience, Clone MP4-25D2, # 16-7048-85)
anti-human IFNg (eBioscience, Clone NIB42, # 16-7318-81)
rabbit anti-human CD3 (Ventana, # 790-4341)
donkey anti-rabbit IgG (H+L) Alexa Fluor 488 (Invitrogen, #A-32790)
chicken anti-mouse IgG (H+L) Alexa Fluor 647 (Invitrogen, #A-21463)
donkey anti-goat IgG (H+L) Alexa Fluor 546 (Invitrogen, #A-32790)

Validation

Antibodies have been validated for use for flow cytometry, immunofluorescence, or functional assays by the manufacturers as stated on their respective websites:

BioLegend: Antibody validation is a critical step in the journey towards obtaining consistent reproducibility in science. To ensure they are both specific and sensitive, we validate our antibodies through a variety of methods including:
-Testing on multiple cell and tissue types with a variety of known expression levels.
-Validation in multiple applications as a cross-check for specificity and to provide additional clarity for researchers.
-Comparison to existing antibody clones.
-Using cell treatments to modulate target expression, such as phosphatase treatment to ensure phospho-antibody specificity.

R&D systems: R&D Systems® takes rigorous steps towards antibody validation and reproducibility. We have been since the beginning. For 30 years, we have used our industry-leading production standards and quality control specifications to develop antibodies that can be relied on for specificity and reproducibility. By developing and testing our products in-house, we can ensure a validated and specific antibody. We are confident in our antibodies and provide 100% guarantee for our products.

Invitrogen: Invitrogen antibodies are currently undergoing a rigorous two-part testing approach
Part 1—Target specificity verification
This helps ensure the antibody will bind to the correct target. Our antibodies are being tested using at least one of the following methods to ensure proper functionality in researcher's experiments. Click on each testing method below for detailed testing strategies, workflow examples, and data figure legends.
-Knockout—expression testing using CRISPR-Cas9 cell models
-Knockdown—expression testing using RNAi to knockdown gene of interest
-Independent antibody verification (IAV)—measurement of target expression is performed using two differentially raised antibodies recognizing the same protein target
-Cell treatment—detecting downstream events following cell treatment
-Relative expression—using naturally occurring variable expression to confirm specificity
-Neutralization—functional blocking of protein activity by antibody binding
-Peptide array—using arrays to test reactivity against known protein modifications
-SNAP-ChIP™—using SNAP-ChIP to test reactivity against known protein modifications
-Immunoprecipitation-Mass Spectrometry (IP-MS)—testing using immunoprecipitation followed by mass spectrometry to identify antibody targets
Part 2—Functional application validation
These tests help ensure the antibody works in a particular application(s) of interest, which may include
(but are not limited to):
-Western blotting
-Flow cytometry
-ChIP
-Immunofluorescence imaging
-Immunohistochemistry
Most antibodies were developed with specific applications in mind. Testing that an antibody generates acceptable results in a specific application is the second part of confirming antibody performance.

Eukaryotic cell lines

Policy information about cell lines and Sex and Gender in Research

Cell line source(s)

Transformed keratinocyte cell line HaCaT was from AddexBio

Authentication

The identity of the cells was verified by the supplier by STR DNA Profiling

Mycoplasma contamination	Passaged HaCaT cells were verified to be mycoplasma free
Commonly misidentified lines (See ICLAC register)	none

Flow Cytometry

Plots

Confirm that:

- [x] The axis labels state the marker and fluorochrome used (e.g. CD4-FITC).
- [x] The axis scales are clearly visible. Include numbers along axes only for bottom left plot of group (a 'group' is an analysis of identical markers).
- [x] All plots are contour plots with outliers or pseudocolor plots.
- [x] A numerical value for number of cells or percentage (with statistics) is provided.

Methodology

Sample preparation	The different methods of sample preparation are described in the material and methods section.
Instrument	Cells were analysed on a FACS LSR II SORP (BD Biosciences).
Software	Data were recorded with BD FACSDiva™ Software v. 6.1.3 and analyzed with FlowJo_v10.7.1
Cell population abundance	The different cell abundances and purities are described in Figures and in the material and methods section.
Gating strategy	The Full gating strategy is shown in Supplementary Figure 8

- [x] Tick this box to confirm that a figure exemplifying the gating strategy is provided in the Supplementary Information.
